# Sensorimnemonic decisions: choosing memories versus sensory information

**DOI:** 10.1016/j.tics.2024.12.010

**Published:** 2025-01-23

**Authors:** Levi Kumle, Anna C. Nobre, Dejan Draschkow

**Affiliations:** 1 Department of Experimental Psychology, University of Oxford, Oxford, UK; 2 Wellcome Centre for Integrative Neuroimaging, Department of Psychiatry, University of Oxford, Oxford, UK; 3 Wu Tsai Institute and Department of Psychology, Yale University, New Haven, CT, USA

## Abstract

We highlight a fundamental psychological function that is central to many of our interactions in the environment – when to rely on memories versus sampling sensory information anew to guide behavior. By operationalizing sensorimnemonic decisions we aim to encourage and advance research into this pivotal process for understanding how memories serve adaptive cognition.

## What are sensorimnemonic decisions?

**Figure 1:**
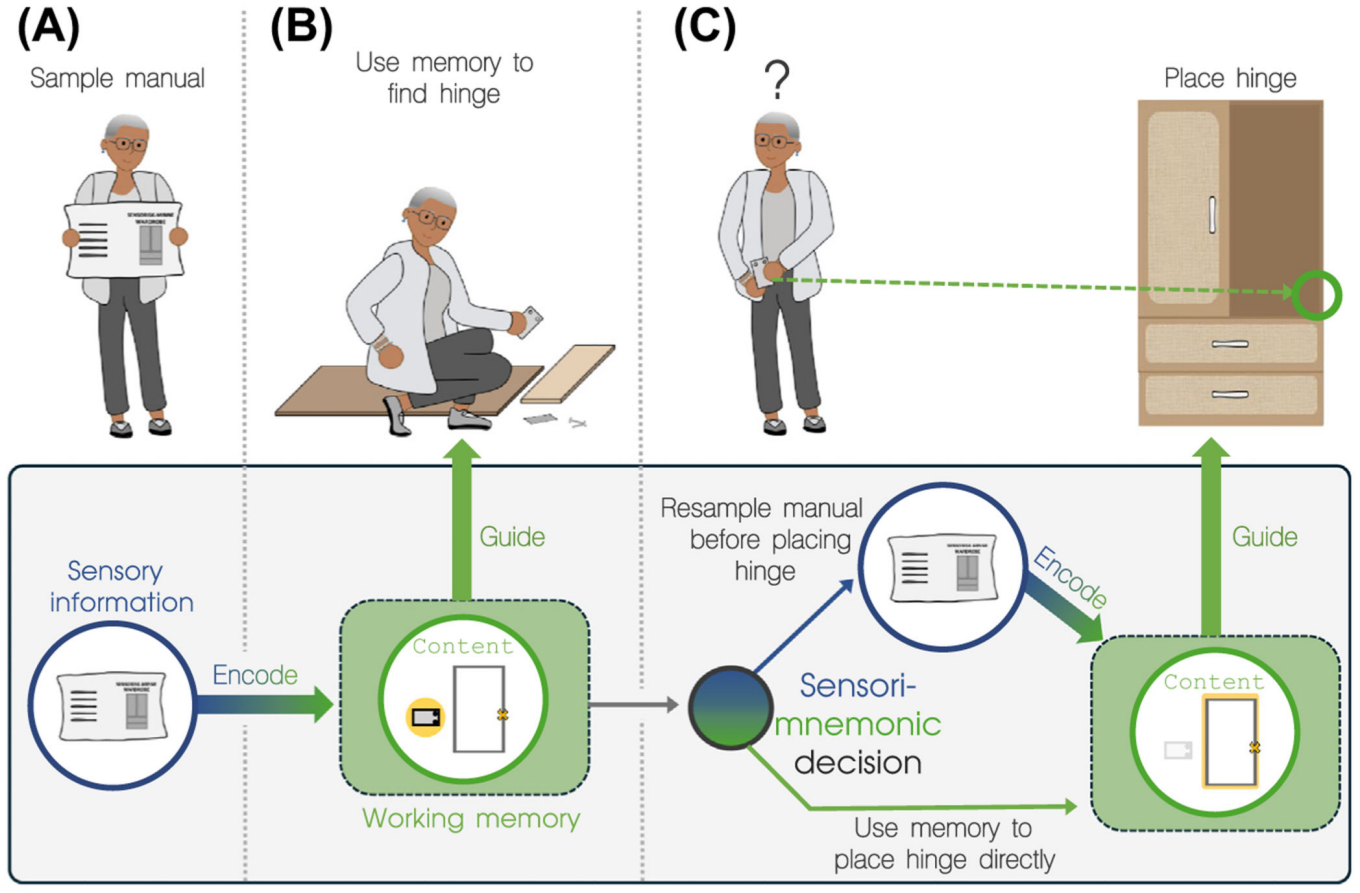
Sensorimnemonic decisions during extended naturalistic behavior. Sensorimnemonic decisions are pivotal during behavioral interactions in extended contexts. Several information-processing stages are relevant to sensorimnemonic decisions. (A) Encoding stage: as goal-directed behavior unfolds over time, there are distinct instances of sensory sampling to encode information into memory. (B) Maintenance stage: once encoded, the information needs to be held in mind and can guide actions (e.g., eye movements during search). (C) Decision stage: critical moments occur when the actor is faced with choosing between memory (use memory to place the hinge directly) and sensory (resample and encode from the manual before placing the hinge) information.

The pivotal moment of choice between relying on memory versus sampling sensory information can be operationalized as a ‘sensorimnemonic decision’ that gates whether and when we use memory to guide natural, free-flowing behavior. These decisions are ubiquitous in every-day cognition and are embedded in the many interactions we have with our environment. For instance, imagine assembling your new wardrobe according to the instruction manual provided. You encode a step from the manual, telling you to find a specific hinge and place it in a designated spot on the wardrobe door (Figure 1A). You proceed to search for the hinge (Figure 1B). After locating it, you face a decision – do you rely on your memory about the specific placement location, or look again at that sensory information from the manual (Figure 1C)? We distill and focus on this central but understudied process. We provide a starting foundation, explore key premises, and highlight open questions concerning the nature of this process and the contextual factors that modulate it.

## Orchestrating the availability of both mnemonic and sensory information

Achieving behavioral goals (placing the hinge screw in our example) requires guidance from both internal memory representations (e.g., the remembered location as specified in the manual) and external sensory information (e.g., the illustration of the wardrobe door in the manual). Orchestrating when to use relevant memory contents versus sampling sensory signals in the service of memory encoding is a major and continual challenge during naturally unfolding behavior. This challenge is heightened by affordances and task demands that evolve in response to our own goals and actions [1]. Yet, our cognitive system seamlessly and constantly chooses whether to use previously encoded signals or sample newly incoming signals. Such sensorimnemonic decisions are intrinsically embedded in processes investigated by researchers in visual action control [1–4] and working memory [5,6]. However, neither field has fully integrated the strengths of the other to address this core aspect of how memories support adaptive cognition.

A key strength of research on visually guided action control is its powerful framework for studying extended behaviors, with a particular emphasis on considering how an evolving task context shapes which sensory information becomes relevant as behavior unfolds. Specifically, a unique advantage is the approach of breaking down behavior into a sequence of action decisions [2,3]. Within these decision arcs, sensory sampling provides relevant information for making goal-directed action decisions and guiding eye, hand, and body movements [1–3]. This framework also allows action decisions to be based on memory information, as well as on a weighted combination of sensory and memory information. For example, using information acquired from prior encounters, memory can guide gaze to likely locations when searching the pile of wardrobe parts (Figure 1B) [2,7]. However, it remains unclear when and how memories are integrated with, or used instead of, sensory information. Further, it is unknown how we orchestrate fundamental memory processes such as encoding, maintenance, and selection of relevant representations to make memories available at the appropriate time and in the appropriate format. In simple terms, the role of ‘mnemonics’ in sensorimnemonic decisions has been insufficiently scrutinized.

In a complementary way, the unique strength of working-memory research lies in its powerful approaches for studying the properties (e.g., type, quantity, quality, confidence, timescales) and processing stages (e.g., encoding, maintaining, selecting, transforming) that are associated with memories [5,6]. However, less is known about when and how memories are deployed to guide actions during temporally extended behaviors [8–11]. This is partly because traditional working-memory tasks rarely offer alternatives to relying on memory, and thus fail to capture situations where individuals can choose between relying on memory and sampling sensory information to guide behavior (Figure 1C). In other words, the ‘sensori’ in sensorimnemonic decisions has received less scrutiny.

## Bringing sensorimnemonic decisions into the focus of research

Combining the strengths of perspectives from action control and working memory allows us to formalize sensorimnemonic decisions as an essential computation that supports when and how memories are used to guide natural behaviors. Critically, by placing sensorimnemonic decisions in the focus of research, several important questions come to the fore. We highlight only a few here. Which factors determine how and when we use memory instead of sampling an accessible external environment? What processing stages precede a sensorimnemonic decision, and what are their consequences? What are the relevant dimensions (e.g., memory properties, visual properties, task demands, motivational aspects, individual differences) of the modulating factors? Which properties of a memory (e.g., type, quantity, quality, confidence, timescale) at which stage (e.g., encoding, maintaining, selecting, transforming) predict this pivotal choice – and how do they interact with the properties of the sensory information? How and when do we shift focus between neural representations linked to memory versus sensory processing to prioritize and prepare the right information at the right time?

Naming and operationalizing sensorimnemonic decisions paves the way for scientific inquiry into the psychological and neural factors that determine when and how we make memories available at the appropriate time to support sensory sampling and action control during extended, free-flowing behavior.

## What shapes the sensorimnemonic process?

Foundational research using average estimates of the trade-off between using memory versus sensory information has highlighted that it is less intuitive than has been assumed [7–11]. For example, having task-relevant content in memory does not guarantee that it will be used to guide behavior [7,10], and estimates of self-determined memory usage are lower than standard memory tasks would predict [8–11]. Further, formative insights into factors that modulate average memory usage – such as the effort required to sample sensory information (e.g., physical movement, time) [8,9] or the reliability of that information [11] – have revealed that the balance between using memory and sampling sensory information for encoding adapts flexibly to task demands. The time is ripe to advance research into the process of choosing between using memory versus sensory information by moving beyond average estimates of memory usage, isolating the relevant information processing stages, and exploring the full range of factors that govern sensorimnemonic decisions.

Here we provide a starting point for investigating key dimensions of interest. Sensorimnemonic decisions will be shaped by factors related to sensory information, memory properties, task demands, motivation, and overall individual differences. For instance, it will be important to investigate how sensorimnemonic decisions are influenced by factors related to the sensory information in the external environment (e.g., feature similarity or familiarity) or factors related to the content in memory (e.g., quality, confidence, timescale) – which have been well studied in traditional memory research [6]. Decision-making and cognitive-effort approaches will be essential for clarifying how the relative utility, value, or cost of external sensory and internal memory signals are weighted to reach sensorimnemonic decisions [1–3,12]. Further, considering participant traits (e.g., actual or perceived memory abilities) will help to reveal how individual differences impact on memory engagement. As such, focusing on sensorimnemonic decisions further links the selective attention [5,13], decision-making [1], cognitive effort [12], and metacognition literatures (Box 1).

Critically, innovative methods will need to be developed to conceptually isolate and empirically investigate separate stages of the sensorimnemonic process. This is particularly important because various factors can systematically influence sensorimnemonic decisions through different stages of the overall process. For example, natural behavior provides flexibility in what we can encode from the sensory environment, and at which point in time we choose to do so. That is, self-determined encoding can set the stage for – but is conceptually distinct from – subsequent sensorimnemonic decisions. While encoding determines which content enters memory, different factors can influence the decision to use that content to guide behavior (decision stage, Figure 1C). For example, distracting hardware on your workbench may not affect how much you encode from the manual (encoding stage) but can lead to you relying less on previously sampled information (decision stage) [9]. Other factors (e.g., effort required to sample sensory information) might instead predominantly affect sensorimnemonic decisions through modulating what and how much is encoded. Last, moving beyond average estimates of memory usage and directly isolating sensorimnemonic decisions will be essential since multiple factors may affect distinct sensorimnemonic decisions differently within the same task context [9]. For example, distracting hardware might only affect your decision to rely on memory while searching for the hinge, but not while placing it.

## Concluding remarks

We highlight sensorimnemonic decisions as a central component in the coordination of memory and sensory information to guide extended behavior. Focusing on this pivotal process will bring multiple fields together, including action control, (working) memory, decision making, selective attention, cognitive effort, and metacognition. Further progress in understanding sensorimnemonic decisions will require embracing the complexities of extended memory-guided behavior and directly operationalizing and isolating the different factors and stages underlying decision points.

## Box 1 Sensorimnemonic decisions and cognitive offloading

A related topic concerning the interplay between sensory and memory utilization is the process of ‘cognitive/ intention offloading’ [14]. Research in this area explores how we strategically use external tools/reminders to substitute holding information in memory (e.g., making a note of the intended location of the hinge).

In ‘intention offloading’ tasks, participants can usually reduce encoding and avoid maintenance by creating external cues (e.g., making a grocery list instead of maintaining the necessary ingredients in memory on the way to the store). By contrast, sensorimnemonic decisions capture the everyday moments in time that require memory to guide behavior but allow the option of resampling external information before doing so (e.g., looking back at the grocery list while searching for items in the store). Interestingly, sampling sensory information – a crucial component of memory encoding – has also been referred to as ‘offloading’. It will be important to reconcile these perspectives in the context of engaging memory.

Among other factors, the ‘offloading’ literature has demonstrated that whether participants decide to rely on unaided memory depends on metacognitive evaluations of their memory abilities and on the value of the information to be remembered [14]. It will be intriguing to investigate whether similar factors impact sensorimnemonic decisions.
